# Analysis of the Fruit Quality of Pear (*Pyrus* spp.) Using Widely Targeted Metabolomics

**DOI:** 10.3390/foods11101440

**Published:** 2022-05-16

**Authors:** Pufan Zheng, Mei Zhang, Xin Fang, Lili Tang, Zhixue Wang, Fuchen Shi

**Affiliations:** 1 College of Life Sciences, Nankai University, Tianjin 300071, China; zhengpufan@foxmail.com (P.Z.); zhangmeinku@163.com (M.Z.); fangxin@mail.nankai.edu.cn (X.F.); lily12tang@gmail.com (L.T.); 2 Institute of Forestry and Pomology, Tianjin Academy of Agricultural Sciences, Tianjin 300384, China; snow3000@foxmail.com

**Keywords:** *Pyrus*, LC-MS, metabolomics, carbohydrate, polyphenol

## Abstract

Pear is a kind of common temperate fruit, whose metabolite composition that contributes to the difference in fruit quality is unclear. This study identified and quantified the metabolites using a widely targeted LC-MS/MS approach in three pear species, including *Pyrus bretschneideri* (PB), *Pyrus usssuriensis* (PU) and *Pyrus pyrifolia* (PP). A total of 493 metabolites were identified, consisting of 68 carbohydrates, 47 organic acids, 50 polyphenols, 21 amino acids, 20 vitamins, etc. The results of PCA and OPLS-DA demonstrated that the metabolite compositions differed distinctly with cultivar variability. Our results also involved some metabolic pathways that may link to the fruit quality based on KEGG pathway analysis, the pathway of phenylalanine metabolism revealed significant differences between PB and PP (*p* < 0.05). Furthermore, the study selected D-xylose, formononetin, procyanidin A1 and *β*-nicotinamide mononucleotide as the major differentially expressed metabolites in the three species. The present study can open new avenues for explaining the differences in fruit quality of the major commercial pear cultivars in China.

## 1. Introduction

Pear (*Pyrus* spp.) is an important cash crop among temperate fruits, belonging to the Rosaceae family [1,2]. The genus *Pyrus* is considered to have derived from the mountains of Southwest China during the Tertiary period 65–55 million years ago and is widely distributed in Asia, Europe and Africa [3,4]. As the third-largest temperate fruit after grape and apple, annual global pear production was as high as 23.7 million tons in recent years, with China, USA and Italy contributing almost 75% of global pear production [5]. Pears are becoming increasingly popular in the consumer market due to their advantages in both taste and nutrition. Other than fresh pears, the pear industry also involves juices, jellies, jams, etc. [6].

As one of the most essential diversity centers for cultivated pears, China has approximately 2000 pear cultivars distributed throughout the country [7]. There are thirteen pear species known to be native to China, the most important of which are *Pyrus bretschneideri* (*P. bretschneideri*), *Pyrus ussuriensis* (*P. ussuriensis*) and *Pyrus pyrifolia* (*P. pyrifolia*) [8,9]. Among them, *P. bretschneideri* and *P. ussuriensis* are the main species grown in the pear-producing regions of northern China, which are also the major pear species of both production and exports in China [10,11]. Generally, the fruit of *P. bretschneideri* has the characteristics of thin skin with waxy luster, and sweet flavor with crisp and juicy flesh. On the other hand, the fruit of *P. ussuriensis* has properties such as high acid content, long-term storage ability, coupled with its flesh being rough and compact. *P. pyrifolia* is cultivated in the high temperature and humid areas of southern China, characterized by large, juicy and is unsuitable for long-term storage [12]. Different cultivars of pear would be favored by different consumers because of their various characteristics in fruit quality.

Fruit quality composed of taste and nutrition as essential components is the main factor determining fruit value and market competitiveness [13,14]. It is popularly believed that the content and proportion of carbohydrates and organic acid are the critical factors to determine the flavor and quality of fruit [15]. The nutritional value of bioactive substances such as anthocyanins, flavonoids and vitamins has become increasingly important with the improvement of public health awareness, which should be considered in the analysis of fruit quality [16,17]. There is a series of complex processes associated with the accumulation of metabolites during fruit ripening. Numerous studies have shown that fruit species (genotype) is the crucial factor determining the composition of metabolites [18,19,20]. However, considerable relevant studies about the metabolites of pear tended to focus on a few specific metabolites, such as carbohydrates [21,22], organic acids [23,24], polyphenols [25,26] and vitamins [27,28]. So far, a comprehensive and systematic investigation of the metabolites variation of different pear species has been needed.

Widely targeted metabolomics provides a relatively fair qualitative and quantitative assessment of the chemical compositions in complex extracts by analyzing a substantial number of metabolites in effective high-throughput techniques [29,30]. In this research, ultra-high performance liquid chromatography tandem quadrupole time-of-flight mass spectrometry (UHPLC-QTOF-MS/MS) was utilized to identify and quantify the metabolites of different pear species, including carbohydrates, organic acids, polyphenols, amino acids, vitamins, etc. This work aimed to clarify the differences in chemical compositions linked to the fruit quality in different pear species, select the major differentially expressed metabolites, as well as provide relevant data for the adjustment and optimization of the cultivar selection in the pear industry of China.

## 2. Materials and Methods

### 2.1. Sampling

The samples of this study were collected in Tianjin, the downstream area of the Haihe River basin of the North China Plain. Tianjin is located in the temperate areas with a semi-humid continental monsoon climate, an annual average temperature of 12.0 °C and an annual precipitation of ~600 mm. There are diverse types of landforms in Tianjin, consisting of plains, mountains, hills, depressions, coastal zones and mudflats. The specific climate and terrain ensure the growth of most deciduous fruit trees in northern China.

In 2019, three species including 9 pear cultivars were collected in the experimental demonstration base (latitude 39.0° N, longitude 116.9° E) of Tianjin Research Institute of Pomology, including PB (*P. bretschneideri* cv. Yuluxiang, *P. bretschneideri* cv. Yali, *P. bretschneideri* cv. Qiubai), PU (*P. usssuriensis* cv. Anli, *P. usssuriensis* cv. Yaguang, *P. usssuriensis* cv. Jieli) and PP (*P. pyrifolia* cv. Qiuyue, *P. pyrifolia* cv. Suisho, *P. pyrifolia* cv. Housi). All the tested species were grafted on the 30-year-old *Pyrus betulifolia*, a common rootstock variety of pear in northern China. The pear trees were grown in an open field with 3 m × 4 m spacing under standard horticultural practices, including disease and pest control. All the tested fruits were harvested at their mature stage, which made them capable of representing some typical features of the species. Ten fruits were collected from four cardinal points in 5 trees with similar growth status as one sample, and 9 samples (3 samples for each cultivar and 3 cultivars for each species) contained 90 fruits were created for each pear species, so as to make the samples as representative of biological variability as possible. The 10 fruits were peeled, pooled and homogenized into one sample using a blender, and all the samples were stored at −80 °C.

### 2.2. Metabolites Extraction

The samples were freeze-dried and ground with liquid nitrogen and 20 mg of the freeze-dried samples was weighted into an EP tube, followed by adding 500 μL extract solution (methanol:acetonitrile:water = 2:2:1 (*V*/*V*), including isotope-labeled internal standard mixture). After vortexing for 30 s, the samples were ground at 35 Hz for 4 min with steel balls, and ultrasonicated in an ice water bath for 5 min. The grinding and ultrasonic treatments were repeated 3 times. After incubating the samples at −40 °C for 1 h, the samples were centrifuged (15 min, 12,000 rpm, 4 °C), and then 250 μL of the supernatant was transferred into a new EP tube for vacuum drying. The dried samples were added to 200 μL of 50% acetonitrile to reconstitute, vortex for 30 s, and ultrasonicate in an ice water bath for 10 min. After centrifuging (15 min, 13,000 rpm, 4 °C), 75 μL of the supernatant was placed into a sample bottle for LC/MS analysis. An equal aliquot of the supernatant for all the samples was mixed to constitute the quality control (QC) sample.

### 2.3. LC-MS/MS Analysis

The LC analysis was conducted utilizing a 1290 Infinity series UHPLC System (Agilent Technologies, Palo Alto, CA, USA). 1 μL aliquot was injected into a Waters ACQUITY UPLC BEH Amide column (1.7 μm, 2.1 × 100 mm, Shanghai, China). The HPLC mobile phase was composed of 25 mmol/L ammonium acetate and 25 mmol/L ammonia hydroxide in water (pH = 9.75) (solvent A) and acetonitrile (solvent B). The elution gradient was 0~0.5 min, 95% B; 0.5~7.0 min, 95~65% B; 7.0~8.0 min, 65~40% B; 8.0~9.0 min, 40% B; 9.0~9.1 min, 40~95% B; 9.1~12.0 min, 95% B. The flow rate was 0.5 mL/min with the column temperature of 25 °C and the auto-sampler temperature of 4 °C.

High-resolution mass spectra data were acquired using a TripleTOF 6600 mass spectrometry (AB Sciex, Framingham, MA, USA) with the information-dependent acquisition (IDA) mode. In this mode, the acquisition software (Analyst TF 1.7, AB Sciex, Framingham, MA, USA) automatically chose ions and collected their secondary mass spectra data based on the primary mass spectra data and preset criteria. In each cycle, the most intensive 12 ions with intensity greater than 100 were selected for MS/MS scanning with a cycle time of 0.56 s. The ion source parameters were as follows: collision energy (CE) as 30 eV; source temperature as 600 °C (TEM); declustering potential (DP) as 60 V; ion spray voltage floating (ISVF) as 5000 V; gas I (GSI), gas II (GSII) and curtain gas (CUR) as 60, 60, and 35 psi, respectively.

### 2.4. Data Preprocessing and Statistical Analysis

MS raw data (.wiff) files were converted to the mzXML format by ProteoWizard (http://proteowizard.sourceforge.net, accessed on 23 October 2021) and processed by the package XCMS in R v3.6.0 (http://www.r-project.org, accessed on 23 October 2021). The process includes peak deconvolution, alignment and integration. Minfrac and cutoff were set as 0.5 and 0.3 respectively. The metabolites were identified using the Biotree in-house MS2 database (Biotree Biomedical Technology Co., Ltd., Shanghai, China; http://www.biotree.cn, accessed on 23 October 2021) within R v3.6.0. Principle component analysis (PCA) was processed using the prcomp function within R v3.6.0. The pie chart for the classification of the metabolites was plotted using OriginPro v9.8 (OriginLab, Northampton, MA, USA). Multiple regression orthogonal partial least squares discrimination analysis (OPLS-DA) was implemented using SIMCA v15.0.2 (Sartorius Stedim Data Analytics AB, Umea, Sweden). The OPLS-DA model was established by validating with a 200× permutation test to avoid the model over-fitting and evaluate the statistical significance of the model.

In order to identify the differentially expressed metabolites, we first used a threshold of variable importance in the projection (VIP > 1) towards the OPLS-DA model to select the metabolites, and then we chose those with a fold change >2 (upregulated) or a fold change <0.5 (downregulated) as the differential metabolites in two paired species. Differentially expressed metabolites were mapped to the database of the Kyoto Encyclopedia of Genes and Genomes (KEGG) pathway (http://www.kegg.jp/kegg, accessed on 23 October 2021) to determine their associated metabolic pathways. KEGG annotation can only find pathways related to the differential metabolites, therefore, comprehensive analyses (including enrichment analysis and topology analysis) of the pathway related to the differentially expressed metabolites were performed using R v3.6.0 to locate the pivotal pathway that were highly correlated with the metabolites’ differences. Student’s *t*-test was used to analyze the accumulation of the differential metabolites. The false discovery rate (FDR) was used to correct the *p*-values for the multiple hypothesis testing correction to reduce the false-positive rate of *p*-values in *t*-test results with large sample size, and the resulting *p*. FDR was used to evaluate the significance of the difference towards the metabolite abundance in the three pear species.

## 3. Results

### 3.1. Chemical Composition Identification

**Figure 1:**
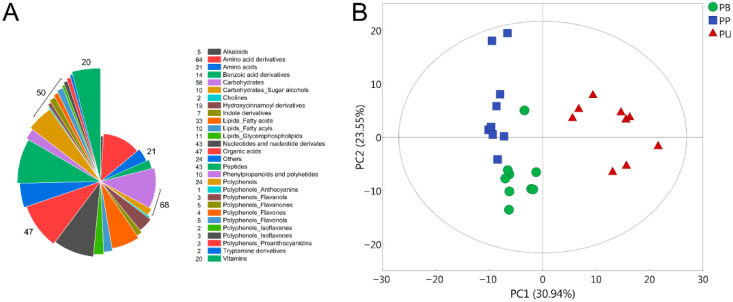
The chemical composition of the three pear species. (**A**) Classification of the 493 metabolites identified from the three pear species. (**B**) PCA analysis of the 493 metabolites in PB, PU and PP. PB, PU, and PP represent *P. bretschneideri*, *P. usssuriensis* and *P. pyrifolia*, respectively.

The widely targeted LC-MS/MS analysis was conducted on the three pear species for the purpose of clarifying the differences in fruit quality among different pear species. A total of 493 metabolites were identified, which contained numerous metabolites that may affect the fruit quality, consisting of 68 carbohydrates, 47 organic acids, 50 polyphenols, 21 amino acids, 20 vitamins and other categories of metabolites (Figure 1A). PCA analysis of the 493 metabolites illuminated that the chemical compositions of the fruits were clearly distinguished on the scatter plot, indicating that there were different metabolite profiles in these three pear species (Figure 1B). Moreover, PU was obviously different from both PB and PP, which might be the underlying reason for the specific fruit quality of PU.

### 3.2. Selection of Differentially Expressed Metabolites

**Figure 2:**
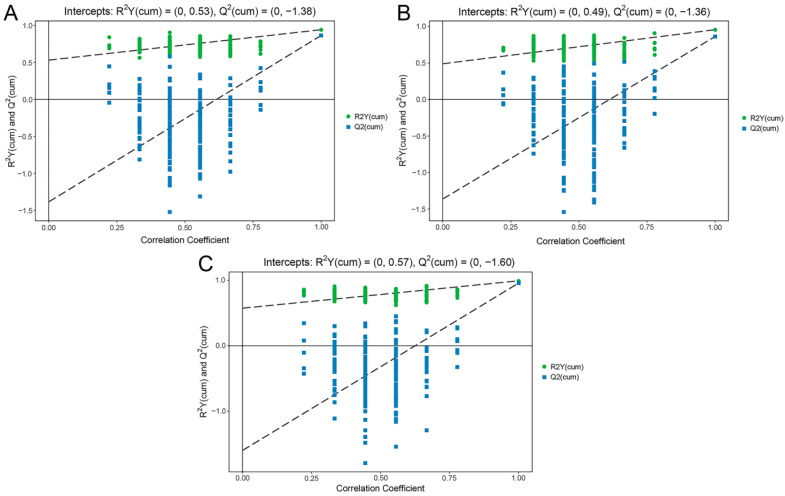
The permutation test results of the OPLS-DA models. The closer the R^2^Y of the original model is to 1, the better the model constructed is consistent with the actual condition of the samples; the closer the Q^2^ of the original model is to 1, the more consistent the distribution will be if new samples are added. (**A**) The model based on PB vs. PU. (**B**) The model based on PB vs. PP. (**C**) The model based on PU vs. PP. PB, PU, and PP represent *P. bretschneideri*, *P. usssuriensis* and *P. pyrifolia*, respectively.

To determine the differentially expressed metabolites of the three pear species, OPLS-DA model was used to perform pairwise comparisons. The permutation test results of the the OPLS-DA models basically illustrated that the original models were stable without over-fitting (Figure 2). In order to avoid the false-positive errors caused by using only one statistical analysis method, we used the variable importance in the projection (VIP > 1) of the OPLS-DA model combined with the fold change (FC > 2 (upregulated) or FC < 0.5 (downregulated)) to determine the differential metabolites.

**Figure 3:**
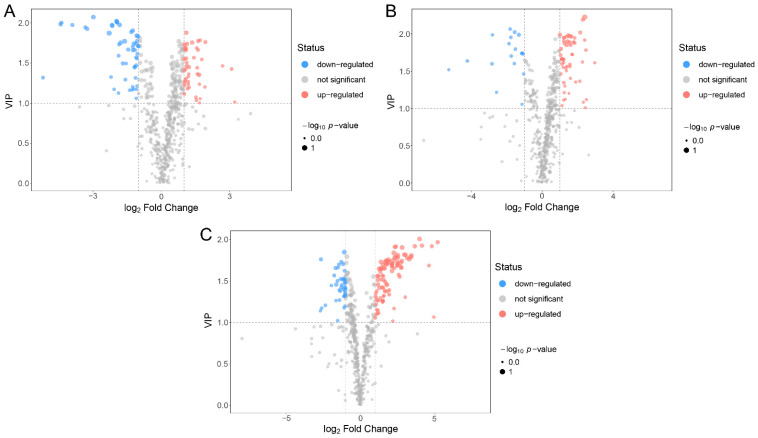
Differences in the metabolites among different pear species. A threshold of variable importance in the projection (VIP > 1) was used to select the metabolites, and then those with a fold change >2 (upregulated) or a fold change <0.5 (downregulated) were chosen as the differential metabolites in two paired species. (**A**) Differentially expressed metabolites between PB and PU. (**B**) Differentially expressed metabolites between PB and PP. (**C**) Differentially expressed metabolites between PU and PP. PB, PU, and PP represent *P. bretschneideri*, *P. usssuriensis* and *P. pyrifolia*, respectively.

VIP reflected the importance and contribution of the variables to the model. Specifically, the greater VIP value indicated that the metabolites differed more markedly between the two groups. In the present study, there were 91 differentially expressed metabolites between PB and PU, 72 differentially expressed metabolites between PB and PP, and 122 differentially expressed metabolites between PU and PP (Figure 3). Among them, 43 metabolites were upregulated, and 48 metabolites were downregulated in PB compared with PU (Figure 3A), 54 metabolites were upregulated and 18 metabolites were downregulated in PB compared with PP (Figure 3B), and 86 metabolites were upregulated and 36 metabolites were down-regulated in PU compared with PP (Figure 3C). The results revealed that PU had a larger number of differentially expressed metabolites upregulated compared with the other two species (48 compared with PB and 86 compared with PP), which possibly led to more a plentiful taste of PU (Figure 3).

### 3.3. KEGG Pathway Analysis of Differentially Expressed Metabolites

We mapped the three groups (PB vs. PU, PB vs. PP, PU vs. PP, respectively) of differentially expressed metabolites to the KEGG database, finding that these differentially expressed metabolites were basically mapped to ‘metabolic pathways’ and ‘biosynthesis’ of secondary metabolites. There were also some metabolites mapped in the pathways that may contribute to the fruit quality, such as flavonoid synthesis, amino acid metabolism, citrate circle, and carbohydrate metabolism. Furthermore, topological analysis and enrichment analysis were conducted on the metabolic pathways of these three groups to define the specific differences in the metabolic pathways in different pear species.

**Figure 4:**
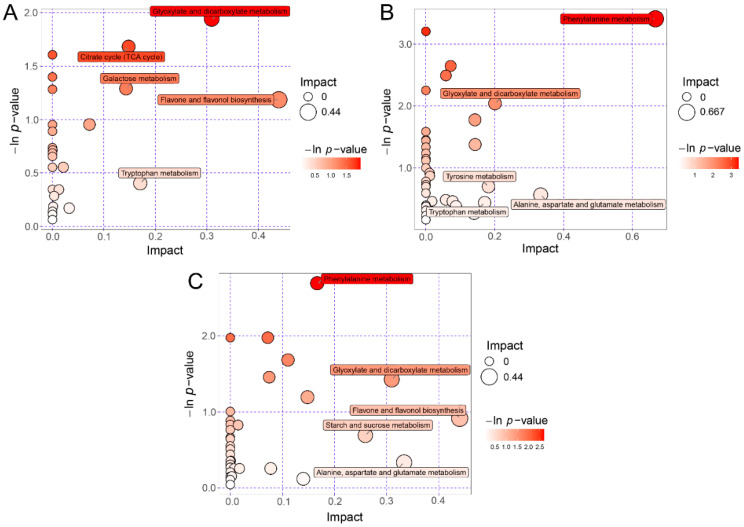
KEGG pathway analysis of differentially expressed metabolites. *x*-axis and the bubble size represented Impact of the pathways in the topological analysis, *y*-axis and the bubble color represented −ln *p* value of the pathways in the enrichment analysis. −ln *p*-value > 3.00 means *p* < 0.05. (**A**) Differential metabolic pathways between PB and PU. (**B**) Differential metabolic pathways between PB and PP. (**C**) Differential metabolic pathways between PU and PP. PB, PU, and PP represent *P. bretschneideri*, *P. usssuriensis* and *P. pyrifolia*, respectively.

Each bubble represented one of the metabolic pathways shown in the bubble plots (Figure 4). The *x*-axis and the bubble size represented the impact of the pathways in the topological analysis, specifically the larger bubble size meant a larger impact. The *y*-axis and the bubble color represented −ln *p* value of the pathways in the enrichment analysis, specifically the darker bubble color meant the larger −ln *p* value (i.e., the smaller *p*). The top 5 pathways were marked based on the Impact score in the topology analysis (Figure 4). The results demonstrated that the differential metabolic pathways of PB and PU were mainly related to flavone and flavonol biosynthesis, glyoxylate and dicarboxylate metabolism, tryptophan metabolism, citrate cycle, and galactose metabolism (Figure 4A). The differential metabolic pathways of PB and PP were substantially associated with amino acid metabolism including phenylalanine, alanine, aspartate, glutamate, tyrosine and tryptophan, especially, the pathway of phenylalanine metabolism showed remarkable differences between PB and PP in the enrichment analysis (−ln *p*-value > 3.00 i.e., *p* < 0.05) (Figure 4B). The differential metabolic pathways of PU and PP basically included flavone and flavonol biosynthesis, alanine, aspartate and glutamate metabolism, glyoxylate and dicarboxylate metabolism, starch and sucrose metabolism, and phenylalanine metabolism (Figure 4C).

### 3.4. Key Compounds Associated with the Fruit Quality

**Figure 5:**
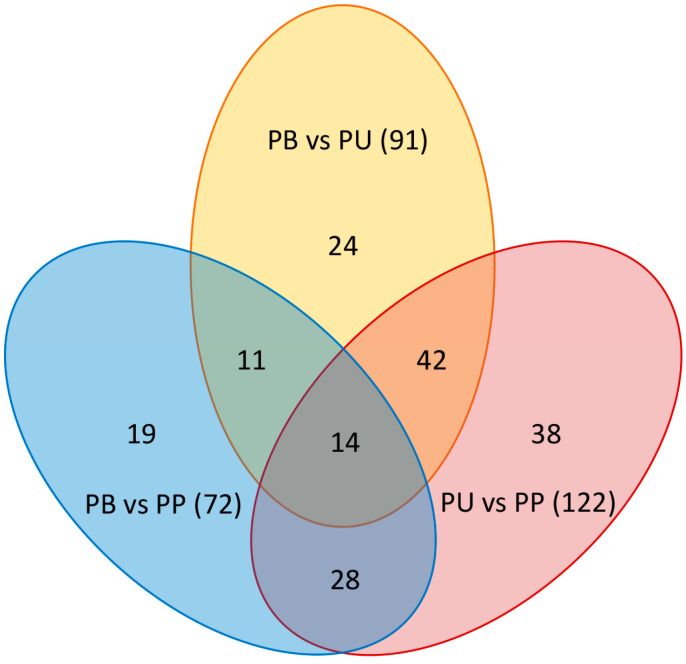
The pairwise comparisons of the three groups of differentially expressed metabolites. PB, PU, and PP represent *P. bretschneideri*, *P. usssuriensis* and *P. pyrifolia*, respectively.

The pairwise comparisons were performed among the three groups of differentially expressed metabolites, and 14 compounds that showed differences in the three species based on VIP value and fold change were screened out (Table 1; Figure 5). These compounds involved carbohydrates, polyphenols, vitamins, etc., which may determine the fruit quality of the pear. The abundance of PU revealed greater than that of PB and PP in 13 differential metabolites (except pantetheine), which might contribute to the specific taste of PU (Table 1). D-xylose was the only carbohydrate obtained from the pairwise comparisons, which may be related to the difference in fruit taste. Formononetin, (−)-naringenin, procyanidin A1, *β*-nicotinamide mononucleotide (vitamin B_3_), acetomenaphthone (vitamin K_4_) and pantetheine (vitamin B_5_), these polyphenols and vitamins with antioxidant activity and biological activity also showed differences in the pairwise comparisons of the three pear species. The significance of the difference in metabolite accumulation was evaluated using Student’s *t*-test and FDR correction. D-xylose, formononetin, procyanidin A1 and *β*-nicotinamide mononucleotide were significantly or extremely significantly different in the pairwise comparisons of fruit metabolites (*p*. FDR < 0.05 or *p*. FDR < 0.01), which could be regarded as the crucial differential metabolites that contribute to the fruit quality of the three pear species.

## 4. Discussion

The widely targeted metabolomics analysis using LC-MS/MS has been successfully applied to various studies of a great number of plant species, such as metabolic regulation [31,32], stress response [33,34], phytochemical analysis [35,36], and cultivar selection [37,38]. Certain kinds of metabolites of pear were involved in previous research, such as carbohydrates [21,22], organic acids [23,24], polyphenols [25,26] and vitamins [27,28], the former researchers had little consideration for the comprehensive and systematic studies on the major metabolites of pear. In the present study, 493 metabolites were identified utilizing LC-MS/MS, consisting of 68 carbohydrates, 47 organic acids, 50 polyphenols, 21 amino acids, 20 vitamins, etc. (Figure 1A). The results of PCA and OPLS-DA suggested that the metabolite compositions of PB, PU and PP were distinctly different, and PU was obviously different from both PB and PP, which provided new evidence for the difference in fruit quality of different pear species (Figure 1B and Figure 3).

Generally, sweetness and acidity are the most important taste indicators of fruit, resulting from both carbohydrate and organic acid, which are proposed to be the determinants of fruit quality [39,40]. A considerable number of carbohydrates and organic acids were observed in our identification results, which is consistent with most previous research [41,42,43]. In this study, the content of D-xylose in PU reported was significantly greater than those in PB and PP (*p*. FDR < 0.05), which may be the potential reason for the unique taste of PU (Table 1). It is worth noting that D-xylose, as a common monosaccharide, has the properties of anti-inflammatory, anti-viral, anti-glycemic and anti-lung cancer, so it even has a certain positive effect on the treatment of COVID-19 [44]. Moreover, no differential metabolites from organic acids were found in the pairwise comparisons, but it was found that the pathway of glyoxylate and dicarboxylate metabolism (glyoxylate cycle), as well as the pathway of citrate cycle (TCA cycle), showed certain differences in the KEGG pathway analysis among the three pear species (Figure 4). Citric acid, malic acid, succinic acid, isocitric acid and other organic acids generated in the metabolic pathways of the glyoxylic acid cycle and TCA cycle are the main sources of the sourness of pear fruits, while the accumulation of acids in fruit cells is the result of several interrelated metabolic processes in different parts of the cells [45,46]. It is worth mentioning that coniferol showed differences in the three groups of fruit metabolites in the pairwise comparisons, and the abundance of PU was significantly higher than that of PP (*p*. FDR < 0.05). As an important precursor for lignin synthesis, coniferol forms lignin through dehydro-oligomerization and further accumulates to form stone cells, which has a critical impact on the fruit firmness and flesh texture of pear [47,48].

As the biologically active nutrients in fruit that are beneficial to health, polyphenols have attracted much attention in recent years, whose composition and abundance are regarded as critical indicators of fruit quality [49,50]. Polyphenols such as flavonoids in fruit have a certain preventive effect on many diseases, including cancer, diabetes, hypertension and cardiovascular disorders as described in earlier observations [51,52]. Among the 50 polyphenols we identified, formononetin, (−)-naringenin and procyanidin A1 were differentially accumulated in the three pear species (Figure 1A; Table 1). Proanthocyanidins are polymerized from flavanols (e.g., catechin and epicatechin) as the basic structural units, they are natural antioxidants with strong antioxidant activity and free radical scavenging activity, which have attracted widespread attention in anti-inflammatory, anti-allergy, anti-aging and improving blood circulation [53,54]. The contents of formononetin and procyanidin A1 in PU were remarkably greater than those in PB and PP (*p*. FDR < 0.05), which may contribute to the broad prospects of PU in the development of health products and medicines (Table 1). Moreover, phenolic compounds are also linked to the astringency, bitterness and flavor of fruit, which is consistent with the sour and astringent characteristics of PU [55].

Most of the vitamins we need are provided mainly through the diet, of which almost all are derived from fruits and vegetables because they cannot be manufactured in the body [56]. Vitamin deficiency may cause a series of diseases, in severe cases, it can even lead to symptoms such as blindness, dementia, scurvy and rickets [57]. We identified 20 vitamins in the metabolites of the three pear species, of these, the concentrations of *β*-nicotinamide mononucleotide (vitamin B_3_), acetomenaphthone (vitamin K_4_) and pantetheine (vitamin B_5_) differed remarkably in these three species (Figure 1A; Table 1). Among them, the abundance of *β*-nicotinamide mononucleotide revealed significant differences in the pairwise comparisons of the three groups, and its accumulation in PU was significantly greater than that in PB and PP (*p*. FDR < 0.05). Nicotinamide mononucleotide (NMN) is a kind of bioactive nucleotide that can be transformed into nicotinamide adenine dinucleotide (NAD) in human cells, which can provide a guarantee for maintaining cell viability, and has outstanding performance in delaying and preventing aging [58].

In addition to the identification and analysis of the metabolites, a certain number of differential metabolic pathways associated with flavonoid synthesis, amino acid metabolism, TCA circle and carbohydrate metabolism were also found in the pear species using topology analysis and enrichment analysis by KEGG. The results of enrichment analysis indicated that the pathway of phenylalanine metabolism responded to remarkable differences between PB and PP (*p* < 0.05; Figure 4). Phenylalanine metabolism is the main source for the synthesis of phenylpropanoid aroma compounds in fruits, and phenylalanine ammonia lyase (PAL) is the vital enzyme in this process [59]. Moreover, phenylalanine metabolism is also a critical metabolic pathway regulating the synthesis of lignin monomers, which is of great significance in the formation of stone cells [60]. Therefore, it is inferred that the phenylalanine metabolic pathway plays a pivotal role in the flavor quality and flesh texture of pear fruit, and the relevant metabolites of this pathway may contribute to the difference in fruit quality between PB and PP.

## 5. Conclusions

This study systematically provided comprehensive information on the composition and abundance of the metabolites in pear using LC-MS/MS analysis. We identified and quantified the metabolites of the three pear species, including carbohydrates, organic acids, polyphenols, amino acids, vitamins and other metabolite classes which may make it desirable in the fruit quality. The results indicated that the metabolite compositions of PB, PU and PP differed distinctly, and PU was obviously different from both PB and PP, which may be associated with the specific taste of PU. The results of OPLS-DA also revealed that PU had a larger number of differentially expressed metabolites upregulated compared with the other two species. In the KEGG pathway analysis, the pathway of phenylalanine metabolism showed remarkable differences between PB and PP (*p* < 0.05), therefore, it was inferred that relevant metabolites of this pathway may be the cause of the difference in fruit quality between PB and PP. We also selected D-xylose, formononetin, procyanidin A1 and *β*-nicotinamide mononucleotide as the major differentially expressed metabolites in the three species, which can be regarded as the important parameters for the quality evaluation of pear with certain directive significance for the pear industry in China.

## Figures and Tables

**Table 1:** Differentially expressed metabolites identified from the pairwise comparisons of the three pear species.

Category	Compounds	Normalized Intensity			*p*. FDR		
		PB	PU	PP	PB vs. PU	PB vs. PP	PU vs. PP
Carbohydrates	D-Xylose	0.253 ± 0.094	1.312 ± 0.435	0.118 ± 0.094	0.005 **	0.042 *	0.001 **
Polyphenols_ Isoflavones	Formononetin	0.199 ± 0.047	0.637 ± 0.172	0.107 ± 0.055	0.006 **	0.019 *	0.001 **
Polyphenols_ Flavanones	(−)-Naringenin	0.268 ± 0.117	0.890 ± 0.593	0.115 ± 0.023	0.058	0.048 *	0.016 *
Polyphenols_ Proanthocyanidins	Procyanidin A1	0.175 ± 0.036	0.515 ± 0.194	0.061 ± 0.048	0.015 *	0.005 **	0.001 **
Vitamins	*β*-Nicotinamide Mono- nucleotide	0.170 ± 0.066	0.673 ± 0.217	0.083 ± 0.063	0.006 **	0.048 *	0.001 **
	Acetomenaphthone	0.128 ± 0.038	0.505 ± 0.180	0.053 ± 0.033	0.006 **	0.070	0.001 **
	Pantetheine	0.310 ± 0.142	0.141 ± 0.081	0.848 ± 0.697	0.012 *	0.106	0.010 *
Hydroxycinnamoyl derivatives	3,4-Dimethoxycinnamic Acid	0.834 ± 0.336	4.061 ± 1.265	0.321 ± 0.331	0.004 **	0.051	0.001 **
	Phenethyl Caffeiate	0.362 ± 0.077	1.019 ± 0.371	0.188 ± 0.099	0.013 *	0.014 *	0.001 **
	Propyl Cinnamate	0.303 ± 0.060	0.948 ± 0.343	0.065 ± 0.025	0.013 *	0.000 **	0.001 **
	Coniferol	0.055 ± 0.032	0.165 ± 0.126	0.026 ± 0.006	0.103	0.111	0.029 *
Amino acid derivatives	3-Amino-3-(4-hydroxy-phenyl) Propanoate	1.296 ± 0.512	6.698 ± 2.071	0.495 ± 0.425	0.004 **	0.025 *	0.001 **
	S-Lactoylglutathione	0.407 ± 0.308	2.824 ± 2.611	0.093 ± 0.044	0.047 *	0.079	0.021 *
Nucleotides and nucleotide derivates	2′-*O*-Methylinosine	0.912 ± 0.351	4.380 ± 1.177	0.291 ± 0.236	0.004 **	0.008 **	0.000 **

The values of the normalized intensity were normalized by internal standard. The mean and standard deviation (SD) values of the normalized intensity are listed. The results of Student’s *t*-test have been corrected with false discovery rate (FDR). * and ** represent the significant differences at *p*. FDR < 0.05 and *p*. FDR < 0.01 levels, respectively. PB, PU, and PP represent *P. bretschneideri*, *P. usssuriensis* and *P. pyrifolia*, respectively.

## Data Availability

The data presented in this study are available on request from the corresponding author.

## Abbreviations

LC-MS, liquid chromatography-mass spectrometry; PB, *Pyrus bretschneideri*; PU, *Pyrus usssuriensis*; PP, *Pyrus pyrifolia*; PCA, principle component analysis; OPLS-DA, orthogonal partial least squares discrimination analysis; KEGG, Kyoto encyclopedia of genes and genomes; VIP, variable importance in the projection; FDR, false discovery rate.
